# Discovery of protein-protein interactions using a combination of linguistic, statistical and graphical information

**DOI:** 10.1186/1471-2105-6-143

**Published:** 2005-06-07

**Authors:** James W Cooper, Aaron Kershenbaum

**Affiliations:** 1 Text Analytics, IBM Thomas J Watson Research Center, PO Box 704, Yorktown Heights, NY 10598, USA; 2 Bioinformatics, IBM Thomas J Watson Research Center, PO Box 218, YorktownHeights, NY 10598, USA

## Abstract

**Background:**

The rapid publication of important research in the biomedical literature makes it increasingly difficult for researchers to keep current with significant work in their area of interest.

**Results:**

This paper reports a scalable method for the discovery of protein-protein interactions in Medline abstracts, using a combination of text analytics, statistical and graphical analysis, and a set of easily implemented rules. Applying these techniques to 12,300 abstracts, a precision of 0.61 and a recall of 0.97 were obtained, (f = 0.74) and when allowing for two-hop and three-hop relations discovered by graphical analysis, the precision was 0.74 (f = 0.83).

**Conclusion:**

This combination of linguistic and statistical approaches appears to provide the highest precision and recall thus far reported in detecting protein-protein relations using text analytic approaches.

## Background

Scientists in molecular biology find that a significant technique for studying protein function is through the study of protein-protein interactions. While the actual experimental study of such interactions remains the most important manner of obtaining these data, the number of protein-protein interactions reported in the literature is substantial and growing rapidly. There are a number of tabulations of these interactions, such as that provided by the Munich Institute for Protein Sequence (MIPS); these tabulations are of necessity incomplete.

To address this problem, we have been developing a group of biology-specific computational annotators that work in conjunction with our group's text analytic software, for the discovery of protein-protein relations in text.

In this paper, we undertook a study that utilizes a combination of computational linguistics, statistics and domain-specific rules to detect protein-protein interactions in a set of Medline abstracts.

The system we describe here is particularly appealing because it can be used both to find known interactions and to find interactions not yet tabulated. According to the National Library of Medicine, Medline contains over 11 million abstracts, with about 40,000 being added each month. Thus, having a scalable, robust system for protein interaction discovery provides a major information tool for molecular biologists.

A number of workers have tackled portions of this problem previously with some partial success. The SUISEKI system [1] recognizes various grammatical frames which may describe protein interactions. They reported high precision (68%) for the shorter patterns and lower precision (21%) for the longer ones.

In a more narrowly focused experiment, Pustejovsky *et. al *[2] described a computational linguistic system for detecting *inhibit *relations, with 90% precision and recall of 57%.

Recently Leroy [3] described Genescene, a software package for detecting relations between genes. They used both rule-based detection and co-occurrence based methods, finding that rule-based relations were 95% correct and co-occurrence based relations 60% correct. Researchers at Ariadne Genomics [4] have quite recently described a system called MedScan, which they report as having 91% precision and 21% recall on human protein-protein interactions.

We [5] have previously described methods for detecting relations between noun phrases and methods for displaying them [6]. In this paper we propose using these techniques along with a combination of statistical and rule-based approaches to identify protein interactions in Medline abstract text. Ideally one would imagine constructing a protein interaction network much like the network that allowed Swanson to discover the relationship between "fish oil" and "Reynaud's disease" [7]. Swanson tabulated terms (chemicals, diseases) that occurred near the initial target name (Reynaud's disease) and formed a network through several papers that eventually led to the conclusion that fish oil was related to the reduction in the symptoms of that disease. The relations extracted in this paper can be used to form just such a network.

This paper discusses the text analytic tools used, and then describes our experiments against a gold standard of protein relations. Finally the results of mining relations across a large set of Medline abstracts are described.

### Text analytic tools

The system used in these experiments is constructed using the TALENT (Text Analysis and Language Engineering Tools) text mining system [8]. The current version of this system is called TafTalent and operates in the Unstructured Information Management (UIMA) environment [9]. It consists of a series of document-level annotators that perform preliminary part-of-speech lookup, tag each word for part of speech, perform a shallow parse of each sentence, and annotate yeast proteins in a manner described below

In this work, the approach is to use tagger and shallow parser annotators primarily for sentence boundary recognition, and use a dictionary annotator derived from public sources to recognize the protein names. The goal of this approach is to be fast and scalable as well as to improve precision and recall over other methods.

After each Medline abstract is processed by the annotators, an annotation consumer program converts these annotations into entries in a database load file. This file contains all of the salient terms per document, their part of speech and their relative token positions in the document. An additional database load file contains the Medline document metadata: dates, titles, authors and ID numbers.

**Figure 1:**
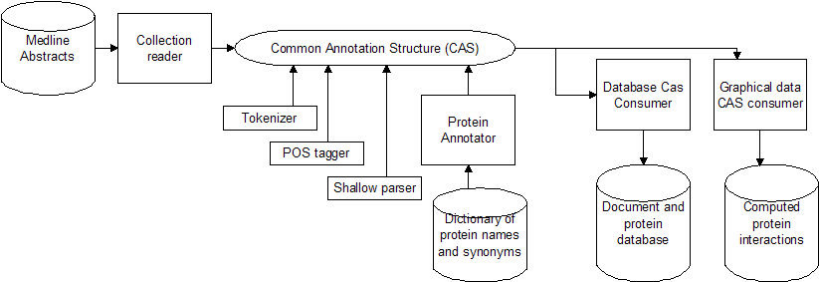
Logical flow of Medline document analysis.

The flow of this process is show in Figure 1. The Medline documents are read one at a time by a collection reader and placed in the Common Annotation Structure provided by the UIMA system. Then a series of sequential annotators tokenize the text, perform part of speech tagging and shallow parsing, and identify the protein names using the dictionary described below. The results are written into a two database tables for further analysis.

Then it is possible to use a few simple database queries to construct a database table of all the unique terms in the document collection, and compute their frequencies, and the number of documents in which they appear once and more than once. Using these data the salience or IQ [10] of each term can be computed.

## Results and discussion

We describe two experiments; one using a small amount of data derived from the MIPS tables on the web, and a larger experiment utilizing 125,000 Medline abstracts.

### Preliminary experiments using MIPS data

The Munich Institute for Protein Sequences (MIPS) maintains a database of published yeast (*saccharomyces cerevisiae*) protein interactions along with a reference to the Medline abstract of the paper in which the interaction is reported. This table contains 2050 protein names and 2604 pairs of protein interactions and provides links to additional information on each protein. The interaction table was parsed and reduced to 959 unique relations, and the protein names and the 564 Medline abstracts downloaded.

An annotator was then developed that compared each lexical token found against the list of proteins and marked those that matched. Then, a simple program was designed to report the location of these proteins within each sentence in each document.

Initially, this was not particularly successful because each protein has a number of possible representations that needed to be matched to a common canonical form. For example, the protein SRV2 can also be represented as Srv2p, SRV2p, CAP and (CAP). Synonyms for most of these proteins are available on pages linked from the original page on the MIPS web site. The dictionary was expanded using these synonyms and the various allowed capitalizations and the analysis rerun, storing all terms and their document positions in a database table.

Even with the expanded protein synonym table, only 388 protein interactions were detected within single sentences that matched those in the MIPS interaction table, and 432 other interactions were detected which did not match those in the MIPS table. This amounted to a precision of 0.47 and a recall of 0.68. Further, there was no particular correlation between the computed strength (mutual information value) of the relation [12] and the likelihood that it agreed with those in the MIPS table.

## Detecting relations in individual documents

In an effort to improve the accuracy of protein-protein interaction detection, a detailed study of 65 of the abstracts was undertaken to determine what algorithms and approaches would be most effective. In this study, each abstract was examined along with a list of the interactions reported by the MIPS table, including all of the synonyms for each protein. This process led to the following conclusions:

1. Some interactions were not reported in the abstracts, but only in the full papers. In fact some review articles contained no protein names at all in the abstracts. This finding is similar to that previously described [1].

2. Some interactions were described that were not tabulated by MIPS. For example, the abstract might mention prior work.

3. Protein complexes were frequently mentioned. For example references are made to dimers such as "Ddc2-Mec1" and trimers such as "Hap2p-Hap3p-Hap5p." Such complexes do, in fact, represent protein interactions and should also be detected and reported.

4. Proteins were frequently referred to by two synonyms separated by a slash, such as "GIM1/YKE2."

5. In all but one case, the interactions were described in the same sentence, and thus resolving co-reference issues would add only marginally to the quality of the interaction detection. Thus, the fact that two proteins occurred in the same abstract, but not in the same sentence was not a good metric for the number of relations we should be able to find.

6. No instances of negation were found.

7. A database query of verbs that lay between two proteins led to the small list shown in Table 1. We note that this list is virtually identical to that determined empirically by previous workers [11].

Accordingly, two additional annotators were written. One annotator recognized protein complexes: dimers and trimers, and the other recognized protein synonyms in the "slash notation" we illustrated in point 4 above. When the annotator found these synonyms, it only annotated one of the two mentions, to avoid skewing the mention statistics. All protein complexes were treated as reports of interactions and annotated as such.

We also annotated the verbs or their noun-equivalents in each sentence, that contained two or more different proteins.

### Evaluation of revised annotations

Examination of protein interactions detected in a few documents showed that nearly all of the relations detected by our proximity algorithm actually existed in the document, whether tabulated by MIPS or not, and that of those our algorithm missed, nearly all were not discussed in the abstract at all.

## Study of a larger set of Medline documents

With these encouraging preliminary results in hand, a study of a larger dataset was undertaken. It is recognized that the initial experiments were on the same data as the rules were developed on, and this second larger set is used to provide a more independent measurement of the accuracy of this approach. In the following experiments, no new protein interaction algorithms were developed, although we did introduce some further filtering to reduce spurious results. The dictionary of protein names and synonyms was the same one derived from MIPS tables. It is our assumption that this is a complete list, though if it is not, the precision and recall numbers would be slightly different.

The query "yeast protein" was submitted against our local indexing of Medline documents through 2002 and a list of the top 12,300 documents was obtained. The MIPS protein interaction table was enhanced by one from Stanley Fields [13]. These documents were annotated as above using the same series of annotators. The same database tables were created from the document ids, terms, and the proteins found in each of them.

The initial results of this experiment returned 912 relations, but only 133 (14%) agreed with the combined gold standard MIPS-Fields table. These and the following results in this section are summarized in Table 2. Considering the large number of abstracts examined, this small number of interactions indicates that the original data referred to by the MIPS table were a serendipitous set which referred specifically to protein-protein interactions. This larger dataset included a number of papers referring to genes which needed to be eliminated from consideration. Modifying the annotator to exclude sentences containing the words "gene," "express," and "encode," improved the accuracy to 17%.

In this larger set of data, protein names may co-occur in more ways that our initial approach allowed for. To reduce the error rate in these experiments, the annotator was further modified to exclude sentences which did not contain one of the verbs in Table 1, or their nominalizations. This resulted in improving the accuracy to 21%.

To further explicate the reasons for the remaining 75% apparent false positives, each relation reported was studied in each abstract where it was detected and conservatively rated either true or false. Of the 343 unmatched relations, 140 additional relations were discovered. While these relations were not in the combined MIPS-Fields table, they were definitely reported in the abstracts. In all questionable cases, the opinion of a biologist was obtained. This leads to 234 out of 437 (53%) relations being discovered correctly.

To further reduce the false positives, sentences containing any negation word (see Table 3) were also excluded from consideration, as were sentences containing the word "allele." It is possible that exclusion of sentences with "not" and the like will also exclude double negatives, but we found only one such case in the entire set of candidate abstracts. This improved the accuracy to 239 out of 381, or 62%.

## Graphical study of secondary relations in the large dataset

We then undertook a study of the graphical relations between proteins, in a similar fashion to that described by Jeong [14]. In this study, we looked at two networks, one of the "true" relations described by the MIPS-Fields table and one described by the network of relations we discovered by text analytic methods. graph to contain only the nodes found in the experimental data.

In our experimental data, we found 385 interactions of which 239 were confirmed by the combined true relations table, while 146 were not, for a precision of 62%. These 385 interactions were among 266 proteins. However, our true relations table contained only 246 of these proteins. Of the 385 interactions found by our approach, 42 involved one of the 20 proteins not part of the true relations table. If we consider only interactions over the 246 proteins common to both tables, we find that 239 of 343 match and 104 do not, for a precision of 70%.

### Results of the graphical study

**Figure 2:**
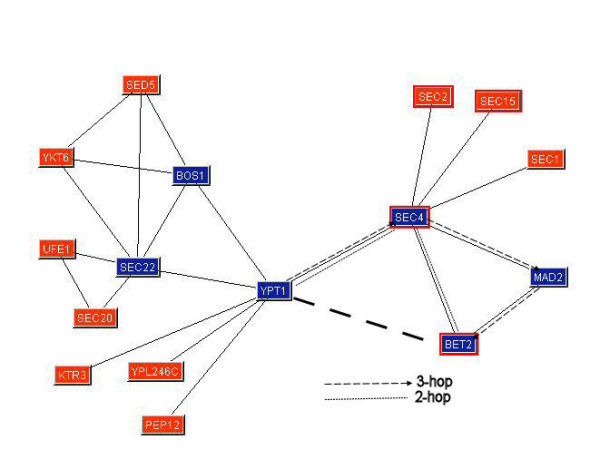
The true relations network around the discovered YPT1-BET2 relation.

In examining the experimental and true relations networks, we built a graph corresponding to each interaction found by our approach but not present in MIPS. We then compared the data to find out if relations which were not directly tabulated in the true relations graph but were found in the experimental data could be explained by indirect relations. For example, in Figure 2, there is no direct relationship between Ypt1 and Bet2 in the true relations network. However, our graphical study discovered such a relationship, and from examination of Figure 2, it is apparent that there is strong support for this relation. There are relations between Ypt1 and Sec4, Bet2 and Sec4, Bet2 and Mad2 and Mad2 and Sec4. Thus, there is a path of length 2 (Bet2-Sec4-Ypt1) and a path of length 3 (Bet2-Mad2-Sec4-Ypt1) between Ypt1 and Bet2. This lends considerable support for the relationship between Ypt1 and Bet2. These 2-hop and 3-hop paths are illustrated in Figure 2 using dotted and dashed lines.

If we then return to our database of documents and mined protein names, the document containing this relation is abstract 1903184, and the supporting text for this relation is:

"We propose that Bet2 modifies Ypt1 and Sec4 in an analogous manner."

Thus, our graphical analysis method discovered an actual relation missed by our text mining system. In this case, it was missed because the verb "modifies" was not one of those we tabulated in Table 1.

### Formal description of algorithm

More formally, given a interaction between two proteins, *P *and *Q*, we define a neighborhood graph, *GN(P,Q)*. We then analyze the cohesion of *GN(P,Q) *for each *P *and *Q *and collect statistics on the cohesion, as described in [15] .

The cohesion of a graph or subgraph is defined as the ratio of the number of edges present to the possible number of edges. In the case of a single node, *n*, in an undirected graph, if the degree (number of incident edges) of *n *is *d *we define the neighborhood of *n *as the set of nodes including the endpoints of these *d *edges, and all edges whose endpoints are in this node set. Say there are *e *such edges. The cohesion, *C(n) *is then defined in Equation (2).



In this paper, we are analyzing the cohesion of a subgraph defined over the union of the neighborhoods of two nodes, specifically *P *and *Q *above. There are also three types of edges in this graph. There are thus many possible definitions of cohesion. For simplicity, we take the conservative approach of only considering 2- and 3-hop paths (i.e., paths between *P *and *Q *which contain 2 or 3 edges).

In the 104 protein interactions found by our method, but not in the combined true relations table, 30 are related by at least one 2-hop path, 34 are related by at least one 3-hop path and 39 are related by at least one 2-hop or 3-hop path.

In 18 of the 30 cases where 2 hop paths were present, more than one path was present, with the average being 2.3, and similarly, 25 of the 34 occurrences of 3-hop paths were multiple occurrences, with the average being 4.1. Since the network contains 30,135 possible pairs, the assumption that these paths support new interactions found by our method seems statistically persuasive. These results are summarized in Table 2.

### Validation of graphical computations

Computation of all relations having 2-hop and 3-hop paths which do not have direct reported interactions gave 30 relations deduced from a combination of 2-hop and 3-hop paths and 9 relations deduced only from 3-hop paths. Of the 2-hop path relations, 15 (50%) of them were found to be true by careful re-examination of the text of the abstracts. This gives us a precision of 74%.

We were unable to discover any abstracts supporting the interactions predicted by the 3-hop relations. However, of the 39 predicted interactions, 20 had 4 or more total 2-hop or 3-hop bridges, and of these 8 were validated by consulting our abstract collection. We suggest that the remaining 12 merit further investigation.

While protein interactions may not be the only reason for this close co-occurrence, other reasons for these graphical relations may be of interest. For example, FAR1 and STE5 are related by 1 2-hop bridge and 6 3-hop bridges. These proteins are reported to be *homologous *[16], that is having structural and functional similarity, and this may also be of significant interest.

## Estimation of recall

Recall, of course, can only be approximated in such a large collection. In the 12,300 document collection, 451 documents were returned as containing one or more of the computed interactions. In reading these documents to validate these interactions, we found only one interaction which was missed by the algorithm because it was referred to across 2 sentences and the co-reference was not resolved by this system.

It is difficult to devise a method for measuring recall when 12,300 documents constitute the sample. We examined 100 randomly chose abstracts from this collection for description of any protein-protein interactions. Since none of the 100 we selected happened to be members of the set of 451 described above, we were looking for additional un-found interactions. None were discovered. On this basis, the recall was effectively 100%.

Since this seemed to be exceedingly optimistic, an experiment was devised which would return the most likely candidate documents where protein relations might have been missed. If we found many missed relations, the recall is reduced.

The method for finding protein interactions we have described above amounts to using a dictionary of yeast proteins names and their synonyms to find co-occurrences of two (or more) such names within a single sentence. Since this amounts to sentence parsing using a well-described tokenizer and parser, followed by simple string matching, and since by direct examination we found almost no cases where the statement of interactions spanned more than one sentence, it is extremely likely that we have found all such cases that exist within the entire 12,300 document collection, and the documents that are returned represent all that actually exist. The recall measurement thus amounts to examining the documents after suitable filtering to find how many actually describe (or do not describe) protein interactions.

Accordingly, we reduced the stringency of our filtering of sentences so that more candidate relations might be discovered. In this experiment, the verb filters (Table 1) were excluded. This approach will return documents containing at least one sentence with two proteins which does not include the word "gene." The other exclusion terms in Table 3 were not used. This resulted in 581 documents, of which 130 were additional to the original set of 451.

These additional 130 abstracts were examined in detail for the description of *any *protein interactions anywhere in the abstract, and 12 such interactions were found. Of these, 2 were discovered across sentence boundaries, requiring anaphora resolution and 2 more occurred in sentences containing the word "gene." This means that 118/130 documents were correctly identified as having no relations, or only 12/130 contained relations, resulting in a recall of at least 90.1%. If we use all 581 abstracts, the recall is much higher (97%). This gives us an F-measure of 0.83.

## Rules used in finding protein interactions

This section summarizes the rules and techniques used in finding the protein interactions.

1. Exclude any sentence containing the terms in Table 3.

2. Recognize proteins from a dictionary of proteins and their synonyms and variant spellings. Exclude all lowercase spellings, which usually represent mutations.

3. Recognize protein complexes by hyphenation.

4. Recognize protein synonyms when separated by a slash.

5. Require any sentence with two or more proteins to contain one of the verbs in Table 1.

6. Allow any sentence containing "form" and "complex" along with two or more proteins.

7. Recognize secondary interactions based on those found by 2-hop and 3-hop connections in the primary table of correct interactions.

## Conclusion

In a large set of 12,300 abstracts, the primary task is filtering out sentences in documents which describe genes and other non-protein interactions. Once this is done, 61% precision is possible, and if the graphical predictions of secondary interactions hold true, the precision is at least 0.74. Based on reading of the abstracts the recall is estimated to be 97%. The f measure is 0.74, based on a precision of 0.61, and is 0.83 based on the precision of 0.74.

These experiments result in respectable precision and considerably higher recall than previously reported methods and tend to indicate that a combination of statistical and linguistic methods [1-4] can give better results than linguistic (frame based) methods alone.

Finally, we note that there is apparently no "silver bullet" to improve detection of protein-protein relations. Instead, the process is one of incremental improvement based on rules and filters of data. However, the set of rules we report here appear to have the highest F-measure yet reported.

## Methods

The MIPS data were downloaded and mined using custom Java programs. Sentence boundaries and tokenization was accomplished using JTalent, a Java wrapper for the TALENT system described above. The noun phrase and verb data were stored in a DB2 database, along with the document number, sentence number and offset, so that the verb co-occurrences listed in Table 1 could be deduced. All of the protein annotators were also written entirely in Java. The graphical network software was also written in Java by AK.

## Authors' contributions

JC carried out the annotator design, text mining and database design. AK carried out the graphical analysis.

## Figures and Tables

**Table 1:** Verbs Used to Describe Protein Interactions

act
activate
associate
bind
complex
co-precipitate
depend
inhibit
interact
mediate
phosphorylate
stabilize

**Table 2:** Summary of precision and recall in recognizing protein interactions under various conditions.

	Matched relations	All relations	Precision	Recall	F measure	Sample size
All sentences	133	912	0.14	1.00 *	0.24	451
Exclude genes	110	660	0.17	1.00 *	0.25	451
Require verbs	94	437	0.21	1.00 *	0.34	451
Discovering relations not in MIPS table	234	437	0.53	1.00 *	0.69	451
Exclude negatives, alleles	239	381	0.61	1.00 *	0.75	451
**Including network analysis data**						
Exclude proteins not part of true relations table.	239	343	0.70	0.97 *	0.81	581
Include only validated 2-hop relations	254	343	0.74	0.97 *	0.83	581

* Assumes that the MIPS table of proteins and synonyms is complete

**Table 3:** Terms that cause a sentence to be excluded from protein interaction discovery

gene
express
encode
no
not
fail
mRNA
transcription
allele

* Assumes that the MIPS table of proteins and synonyms is complete
